# Interrupted Access to and Use of Family Planning Among Youth in a Community‐Based Service in Zimbabwe During the First Year of the COVID‐19 Pandemic

**DOI:** 10.1111/sifp.12203

**Published:** 2022-06-22

**Authors:** Constancia V. Mavodza, Sarah Bernays, Constance R.S. Mackworth‐Young, Rangarirayi Nyamwanza, Portia Nzombe, Ethel Dauya, Chido Dziva Chikwari, Mandikudza Tembo, Tsitsi Apollo, Owen Mugurungi, Bernard Madzima, Katharina Kranzer, Rashida Abbas Ferrand, Joanna Busza

**Affiliations:** ^1^ Biomedical Research and Training Institute Harare Zimbabwe; ^2^ Department of Public Health, Environments and Society, Faculty of Public Health and Policy London School of Hygiene and Tropical Medicine London UK; ^3^ Department of Global Health and Development, Faculty of Public Health and Policy London School of Hygiene and Tropical Medicine London United Kingdom; ^4^ School of Public Health University of Sydney Sydney Australia; ^5^ Clinical Research Department, Faculty of Infectious and Tropical Diseases London School of Hygiene and Tropical Medicine London UK; ^6^ MRC London School of Hygiene and Tropical Medicine London UK; ^7^ Ministry of Health and Child Care HIV and TB Department Harare Zimbabwe; ^8^ National AIDS Council Harare Zimbabwe; ^9^ Division of Infectious and Tropical Medicine Medical Centre of the University of Munich Munich Germany

**Keywords:** COVID‐19, youth, contraceptive access, resilient systems, Zimbabwe

## Abstract

The COVID‐19 pandemic has had serious impacts on economic, social, and health systems, and fragile public health systems have become overburdened in many countries, exacerbating existing service delivery challenges. This study describes the impact of the COVID‐19 pandemic on family planning services within a community‐based integrated HIV and sexual and reproductive health intervention for youth aged 16–24 years being trialled in Zimbabwe (CHIEDZA). It examines the experiences of health providers and clients in relation to how the first year of the pandemic affected access to and use of contraceptives.

A total of 72 interviews were conducted at four time points between April 2020 and May 2021 with CHIEDZA providers and clients. Nonparticipant observations of the CHIEDZA centers (n = 18) and participant observations of provider and research team meetings (n = 14) during this time were also documented. All interviews, field notes, and meeting minutes were thematically analyzed using NVivo, word processing and analytical memos.

We illustrate how the CHIEDZA intervention functioned before COVID‐19 and then chronologically track the effects of the pandemic on access to and use of family planning methods during the temporary closure, subsequent reopening, and adaptation of the intervention in response to the pandemic. We show how existing barriers to family planning access were exacerbated by the pandemic and how youths navigated and responded to these barriers. Fear of contracting COVID‐19 and the consequences of breaking national lockdown restrictions hindered youths’ access to services, leading some to discontinue using contraceptives. This study highlights the critical need for quality youth‐friendly services, which was heightened by the conditions of a pandemic. The study demonstrates that the uneven protection and prioritization of some sexual and reproductive health services (e.g., HIV treatment) over others (e.g., family planning) reflects an investment in only narrow components of the health system, which undermines broader systemic resilience. Additionally, we explore learning about health system vulnerabilities more broadly and the strong need for investment in sustainable and resilient health systems and comprehensive sexual and reproductive health services for youth. We highlight the role, gaps, and opportunities for an intervention such as CHIEDZA operating in community settings but distinct from the health system during the pandemic.

## INTRODUCTION

Youth aged 15–24 years are at high risk of poor sexual and reproductive health (SRH) outcomes (WHO 2011, 2017). Sub‐Saharan Africa (SSA) has the world's highest rates of adolescent pregnancies (10–19 years), with 28 percent and 25 percent of girls in West and Central Africa and in East and Southern Africa, respectively, having given birth by age 18 (Loaiza and Liang 2013). Additionally, approximately 35 percent of pregnancies among 15‐ to 19‐year olds are unintended, underscoring an unmet need for family planning among young women (Chae et al. 2017).

In Zimbabwe, there is a high unmet need for family planning among young women aged 15–24 years. The most recent demographic health survey (DHS) data in 2015 showed that the unmet need for family planning among women of reproductive age (15–45 years) was 12.6 percent and was particularly high for unmarried young women: 37 percent (15–19 years) and 17 percent (20–24 years) compared to married young women in the same age groups, 12.6 percent and 10 percent, respectively (Zimbabwe National Statistics Agency and ICF International 2016). According to the 2019 Multiple Indicator Cluster Surveys Report, 17.6 percent of 15‐ to 19‐year olds and 24.1 percent of 20‐ to 24‐year olds gave birth and teenage fertility rates are 108 (15–19 years) and 193 (20–24 years) births per 1,000 girls, respectively (Zimbabwe National Statistics Agency (ZIMSTAT) and UNICEF 2019).

Despite the country's high modern contraceptive prevalence rate (65 percent), challenges in ensuring youth‐friendly, voluntary, informed choice and access to a range of contraceptive methods for youth remain in the Zimbabwean health care system (Ministry of Health and Child Care 2016; Zimbabwe National Statistics Agency and ICF International 2016). Most people (73 percent) access family planning services from the public sector, which has facility‐based services and community‐based distribution (CBD) programs. According to the *Zimbabwe National Family Planning Costed Implementation Plan 2016–2020*, facility services should offer the full method mix, but they are inadequately equipped, the services are not free, and there are not enough skilled personnel to provide long‐acting reversible contraceptives (LARCs). The CBD program offers information on all contraceptive methods but only provides pills and condoms in the community, and coverage has been declining over the years (Ministry of Health and Child Care 2016). Access to family planning information for young people has also been limited, with only 13 percent of 15‐ to 19‐year olds having media access to family planning information, compared to 24 percent of the rest of the population, and only 3 percent of them reported receiving family planning information at either outreach or static clinics (Ministry of Health and Child Care 2016). The government of Zimbabwe pledged to invest in and address the unmet need for family planning among married adolescents aged 15–19 years and to reduce it from 12.6 percent to 8.5 percent by 2020 (Govt. of Zimbabwe 2017).

Before the COVID‐19 pandemic, Zimbabwe's health system was fragile, with degraded infrastructure and shortages of basic health supplies and staff (Kidia 2018; Green 2018; Meldrum 2008), largely due to the economic crisis that began in the early 2000s. Since then, user fees have increased sharply (Makoni 2020; Green 2018), and national shortages of contraceptive commodities and devices are frequent (Manyonga 2019; Moyo 2019). Since the onset of the COVID‐19 pandemic in March 2020, programmers and researchers have highlighted and/or predicted that the pandemic would impede youth's access to SRH globally, leading to negative health and social outcomes (Mmeje, Coleman, and Chang 2020; Wilkinson, Kottke, and Berlan 2020; Lindberg, Bell, and Kantor 2020; Lewis et al. 2021b; Compact for Young People in Humanitarian Action 2020; UNFPA 2020, 2021; Hussein 2020; Both, Castle, and Hensen 2021). Real‐world data confirming these predictions have started to emerge. As elsewhere, the onset of the COVID‐19 pandemic resulted in disruptions to contraceptive supply chains in Zimbabwe (Aly et al. 2020; Kumar, Malviya, and Sharma 2020). As part of COVID‐19 mitigation measures, the government, private sector, and NGOs were forced to close health facilities, mobile clinics, and community‐based interventions (Riley et al. 2020; Pratt and Frost 2020), reducing access to health services.

This study aims to explore the role of the COVID‐19 epidemic and associated mitigation measures in shaping access to and use of contraceptives over time by young women within a cluster randomized trial evaluating a community‐based integrated HIV and SRH intervention in Zimbabwe.

## METHODOLOGY

### Study Design

This study was part of the nested process evaluation for the CHIEDZA trial, which uses the Medical Research Council's guidance framework to explore and understand the implementation, mechanisms of change, and context of this multicomponent trial (Moore et al. 2015). A qualitative, exploratory and descriptive design was used for this study to explore the perceptions and experiences of CHIEDZA health providers in providing family planning services and youth clients in accessing and using contraceptives during the first year of the COVID‐19 pandemic. A qualitative approach was chosen because it permits information sharing between researchers and participants, allowing for in‐depth exploration of experiences (Khan and Chovanec 2010).

### Study Setting

Zimbabwe has a population of approximately 15.2 million people, with a median age of 18.7 years, and 38 percent reside in urban areas (“Worldometer” 2022). Young people aged 15–24 years make up 20 percent of the population, with 42 percent of women of reproductive age and 34 percent of maternal deaths also being within this age group. Contraceptive use among adolescents, both married and unmarried, is 46 percent compared to the national average of 67 percent (Zimbabwe National Statistics Agency and ICF International 2016). According to the 2015 DHS, among women aged 15–19 years, 10 percent (Harare), 12 percent (Bulawayo), and 25 percent (Mashonaland East) had begun childbearing. The unmet need for family planning among women of reproductive age (15–49 years) was 10 percent in Harare and 9 percent in both Bulawayo and Mashonaland East (Zimbabwe National Statistics Agency and ICF International 2016).

### Family Planning in the CHIEDZA Intervention

CHIEDZA is a cluster randomized trial testing a comprehensive and integrated intervention of HIV and SRH services for youth (16–24 years) delivered in community‐based settings in three provinces in Zimbabwe: Harare, Mashonaland East, and Bulawayo. The services are delivered by a team consisting of nurses, community health workers, youth workers and a counsellor. Each province has four intervention clusters and four control clusters (Dziva Chikwari et al. 2022). A key component of CHIEDZA is the use of some clients as community mobilizers, tasked with reaching out to youths and sensitising communities to the intervention to increase engagement.

As part of the package of CHIEDZA services, youth‐friendly trained providers offer family planning information and a choice of methods, including condoms. Nurses dispense oral contraceptives and Depo‐Provera (Depo) injectibles for young women, and community health workers distribute condoms at the community centers located in the intervention clusters. All commodities are provided free of charge. At the trial's inception, implants and intrauterine devices (IUDs), LARCs, were offered via referral to a nongovernmental organization, Population Services Zimbabwe (PSZ), at either public sector clinics or specific PSZ centers. This changed in October 2020 when CHIEDZA partnered with PSZ to offer these methods at the CHIEDZA centers alongside the other methods. CHIEDZA was implemented in the absence of affordable alternatives, and access centered on making contraceptives easily available both physically and economically.

### Study Context

In September 2019, Zimbabwe experienced a national shortage of contraceptive commodities (Moyo 2019; Manyonga 2019), which lasted approximately one year, thus preceding and then overlapping with the first wave of the COVID‐19 pandemic. During this period, in October 2019, CHIEDZA went from offering three months of supply of oral contraceptives (per national guidelines) to one month of supply to avoid stockouts. This study focuses on the COVID‐19 impact during the first year of the pandemic: March 2020 to May 2021.

When a national state of emergency was declared on March 28, 2020, in response to the COVID‐19 pandemic and a level 5 restrictive national lockdown began on March 31, 2020, there were already depleted supplies of family planning commodities across Zimbabwe. During this lockdown, HIV care and treatment services were considered essential services and could be accessed at selected public and private facilities that remained open. HIV testing and other SRH services, such as family planning, were not considered essential services and could not be readily accessed during this time. This lockdown lasted six weeks until May 14, 2020 and impeded the provision of a wide range of non‐COVID‐19 health services. In May 2020, the lockdown was reduced to level 2, during which businesses and other essential services were allowed to reopen COVID‐19 protective measures in place (Table 1). The beginning of the level 5 lockdown coincided with school closures for the holidays at the end of March 2020. The Ministries of Education determined whether schools would open, and the school schedule remained disrupted in the first year of the pandemic. In the instances when schools reopened, attendance was prioritized for classes that had to sit for national exams in October–December 2020 (Dziva, Zhou, and Zvobgo 2021).

**TABLE 1:** Summary description of the two lockdown levels that Zimbabwe underwent during the first year of the COVID‐19 pandemic sourced from Public Health (COVID‐19 Prevention, Containment and Treatment) (National Lockdown) Orders in Zimbabwe

**Lockdown levels**	**Description**
Level 5	The entire country is in national lockdown. All businesses closed, except essential services (as defined by the statutory instrument). Those working in essential services were able to leave home for work but required to carry documentation to prove employment. Public and private clinics had restricted operating hours. Mobility restrictions—stay‐at‐home orders in place for all nonessential workers. Travel for necessities (groceries) limited to 5 km radius, except if seeking medical attention.
Level 2	Formal businesses are allowed to reopen with COVID‐19 prevention measures (mask‐wearing, sanitising, testing of employees) in place. Movement was allowed beyond the 5 km radius, provided one had a valid letter to show the reason/rationale for this movement. Mandatory wearing of masks in public. Police presence is used to monitor movement and ensure everyone is wearing masks.

**FIGURE 1:**
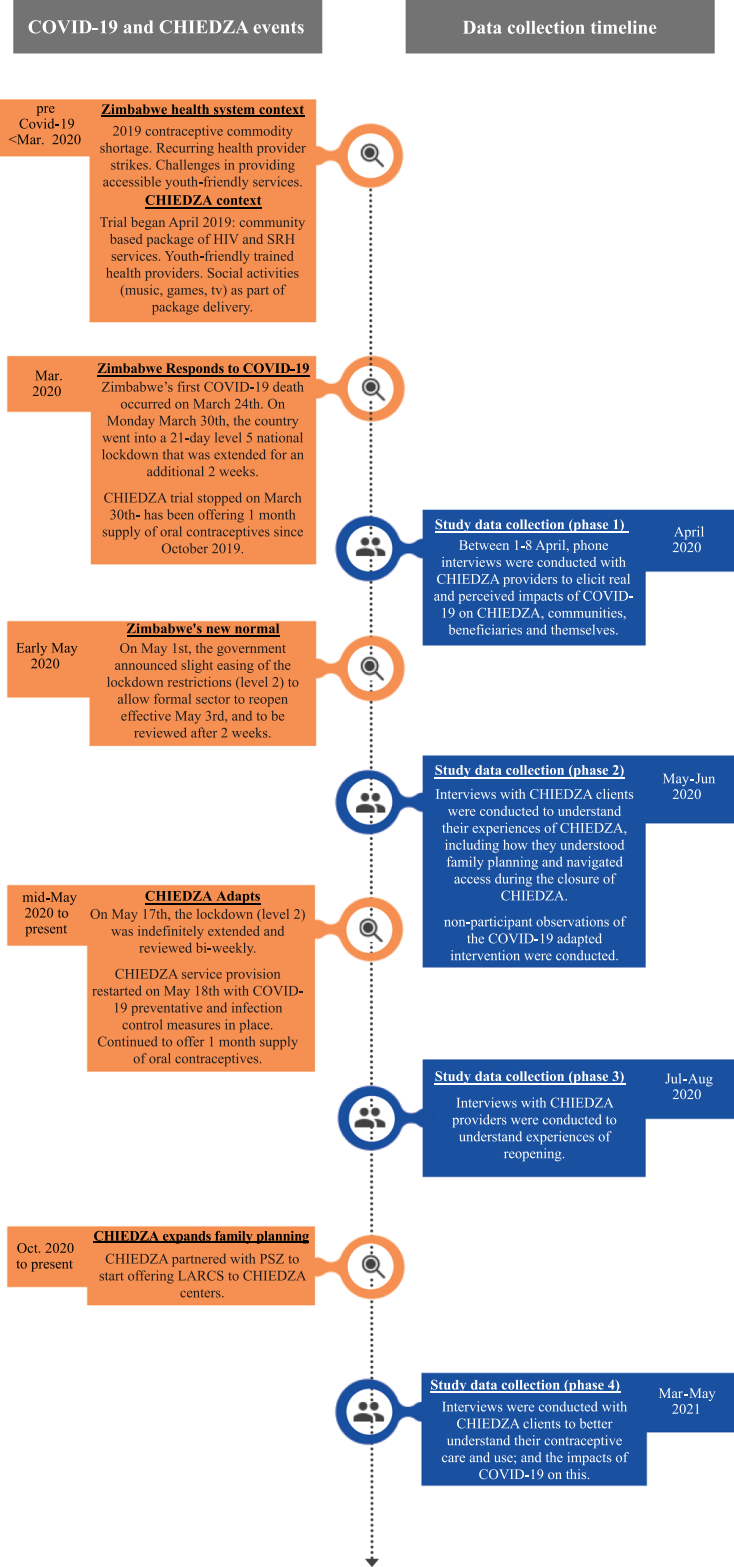
COVID‐19 in Zimbabwe, CHIEDZA implementation and data collection timelines for this study

At the onset of COVID‐19, the CHIEDZA trial closed for the six weeks of the level 5 lockdown. The trial reopened on May 18, 2020 as it was granted an essential service exception under level 2 lockdown. COVID‐19 infection prevention control (IPC) measures were implemented to ensure safe service provision. Some of these measures changed the structure of service delivery. The structural adaptations included moving service delivery from inside community centers to an outdoor space within the centers’ compounds, reducing service delivery hours to accommodate COVID‐19 curfew restrictions, removing social activities, and halting mobilization activities in the community (Figure 1).

### Data Collection

This study draws on 72 interviews conducted over four phases with CHIEDZA health providers and youth clients from all twelve intervention clusters in the three provinces (Figure 1; Table 2).

**TABLE 2:** Qualitative data collection timelines, participants, and methods

**Phase**	**Sampling strategy**	**Type of participants**	**Data collection method**	**Objective**
1. April 2020	Purposive sample: each province and each type of healthcare provider represented	16 health providers (10 females; 6 males)	Phone interviews	To understand how the advent of COVID‐19 and lockdown conditions affected the provision of health services for young people who depended on CHIEDZA services
2. May–June 2020	Purposive sample: all the youth mobilizers to get representation by sex and cluster. Nonmobilizers for representation of ordinary clients	26 youth clients (15 females; 13 males) (23 youth community mobilizers: 3 clients)	3 phone interviews 23 in‐person interviews	To understand clients’ experience of the adapted CHIEDZA intervention, how these adaptations influenced client interactions with the intervention, and, how clients navigated SRH health care during the COVID‐19 pandemic
3. July–August 2020	Purposive sample: each province and each type of healthcare provider represented; 7 repeat interviews to examine change in perception over time	15 health providers (10 females; 5 males)	5 phone interviews 10 in‐person interviews	Explore providers’ experiences of providing COVID‐19‐adapted CHIEDZA health services and their perceptions of health impacts of the lockdown on young people coming to CHIEDZA
4. March–May 2021	Purposive sample: for maximum variation by contraceptive type they use (short‐acting and long‐acting)	15 female youth clients All in‐person interviews	8 narrative‐style interviews 7 interviews with topic guide	To understand clients’ experience of the adapted CHIEDZA intervention, how these adaptations influenced client interactions with the intervention, and, how clients navigated SRH health care during the COVID‐19 pandemic‐a year into the pandemic examine clients’ narratives and experiences of contraceptive access and use as well as the impacts of COVID‐19 on these
May–July 2020 Mar‐Apr 2021	Convenience and purposive: all provinces included. Easy to travel to and from.	18 nonparticipant observations events	10 observations CHIEDZA reopened 8 observations a year into pandemic	To witness how CHIEDZA was now being implemented and received after the level 5 lockdown Examine the impact of the pandemic on the intervention, a year into the pandemic
March 2020–May 2021	All debrief meetings during this period	14 participant observations	Meeting minutes	To describe and understand the study team and provider experiences and perceptions of providing family planning services within an adapted intervention.

### Provider Interviews

We interviewed 16 providers (Phase 1) at the beginning of lockdown and 15 providers (Phase 3) after CHIEDZA had reopened as an essential service (Table 2; Figure 1). Telephonic interviews were conducted in Phase 1 due to lockdown mobility restrictions. Health providers were invited to participate in phone interviews during the last team meeting before the lockdown went into effect. Twenty providers signed written consent forms, and 16 were interviewed. Of the remaining four, two did not respond when contacted for a phone interview, one represented a health provider cadre (Community Health Worker) that had been well‐represented already, and one was working on an intermittent volunteer basis and therefore excluded from these interviews.

Eight weeks after CHIEDZA reopened, an additional 15 provider interviews were conducted in Phase 3. Seven of these were repeat interviews, following up the same providers interviewed in Phase 1 to examine whether and how provider perceptions of COVID‐19 and CHIEDZA had changed over the two data collection time points. The Phase 3 interviews were conducted either in person or by phone, depending on the national social distancing and intercity travel restrictions at the time of each interview. A research assistant in the provinces we could not travel to obtained written consent before the telephonic interviews were conducted by CM. The semistructured interviews were conducted using a topic guide (see the Supporting Information) to explore the objectives under investigation (Table 2).

### Client Interviews

Interviews were conducted with 26 CHIEDZA clients (Phase 2) immediately after CHIEDZA had reopened in May 2020, with an additional 15 clients (Phase 4) interviewed about a year after the start of the pandemic in March 2021 (Table 2). In Phase 2, all 23 of the CHIEDZA youth community mobilizers were invited to participate in the study because of their combined experience of being young people, clients of CHIEDZA, and a link to communities where CHIEDZA services occur. All (n = 23) agreed to participate and provided written consent, and interviews occurred either in person or telephonically. For those who chose telephone interviews, written consent was obtained by a research assistant, and interviews were conducted at a time convenient for the mobilizers. Additional interviews (n = 3) were conducted with CHIEDZA clients, who were not mobilizers, to explore any differences/similarities between mobilizers and nonmobilizers in CHIEDZA experiences: no major differences were seen. Topic guides (see the Supporting Information) were used for the semistructured interviews.

In March 2021, 12 months after COVID‐19 was declared a pandemic, Phase 4 interviews (Figure 1) were conducted with female CHIEDZA clients using contraceptive methods (n = 15). Unstructured interviews were used with some of the clients (n = 8) to elicit their narratives about their SRH, including pregnancy prevention, perceptions of fertility, contraceptive use/uptake and the impacts of COVID‐19 on these issues (Table 2). The unstructured style enabled us to collect data that were specifically oriented toward exploring the concerns and issues selected by young people. Although there was flexibility in the topics discussed in the prior rounds of data collection, an unstructured interview approach was adopted to reflect young people's priorities rather than those deemed relevant by the researchers. In the remaining interviews with seven clients, a topic guide was used to elicit their experiences of contraceptive access and use.

### Observations

Nonparticipant observations (n = 18) were conducted at 10 of the 12 CHIEDZA centers over two time periods (Table 2) using observation guides (see the Supporting Information).

Participant observations of meetings between the research and implementation (CHIEDZA providers and coordinators) teams’ meetings (n = 14) that occurred monthly between March 2020 and May 2021 were also conducted. An experienced qualitative researcher (CM) was part of these meetings as both a contributor to the team discussions (participant role) and a data collector observing and taking notes during proceedings.

### Data Analysis

All interviews were audio‐recorded and conducted in English, Shona, or Ndebele depending on participant preference and subsequently transcribed in English. The interviews ranged in length between 15 and 90 minutes, lasting approximately 45 minutes on average. Observation and meeting notes were written up using guiding templates. All 72 transcripts were read, and emerging inductive themes were compiled in data summary notes using Microsoft Word. Following the data summaries, the drafting of analytical memos, which explored connections between codes and developed emerging ideas (Birks, Chapman, and Francis 2008), and NVivo were employed to advance the analysis. Coded excerpts were extracted from the transcripts and data summaries and grouped under the identified themes. The analytical processes were iterative and occurred during each data collection phase with phase–phase comparison of emerging themes. The process involved collaborative discussions of codes and emerging themes among CM, SB, and JB.

The group of health care providers who participated was small, and some professional roles were represented in this study by only one person. To protect anonymity, quotes from providers are labeled by interview number, sex, province, and time point of data collection. Quotes from CHIEDZA clients are labeled by interview number, province, sex, age, and time point of data collection. PSZ offers free LARCS at the CHIEDZA centers (through partnership) and offers family planning methods at their own centers and public facilities. To delineate between the two in participant quotes, “non‐CHIEDZA” will be used to refer to the latter scenario, and “CHIEDZA” will be used to refer to the former.

### Ethical Approval

Ethical approval was granted by the Medical Research Council of Zimbabwe (MRC/A/2266), London School of Hygiene and Tropical Medicine (14652), and Biomedical Research and Training Institute (AP144/2018). All participants provided written informed consent.

### Findings

This section first presents findings to illustrate how the CHIEDZA intervention functioned before COVID‐19 and then describes the effects of the pandemic on access to and use of family planning methods during the intervention's temporary closure, subsequent reopening, and adaptation of the intervention in response to the pandemic.

### It Was Already Hard: Difficult Access to SRH Services Preceded COVID‐19

A significant portion of the family planning access challenges that youth in Zimbabwe faced before COVID‐19 were due to the already fragile health system and worsening socioeconomic situation in the country. For example, CHIEDZA providers explained how accessing LARCs at other health programs and clinics came at a financial cost for youths.

The clinic will charge money for LARCS. PSI charges but it is a smaller amount. And PSZ [non‐CHIEDZA] offers for free when commodities are available… (CHIEDZA provider‐IDI11, female, Mashonaland East, Phase 3).

In the months before the pandemic, at times some public sector facilities did not have enough commodities to provide youths with the free family planning method of their choice. To try to circumvent commodity shortages, these facilities referred young women to an NGO partner, which also presented access challenges.

So sometimes clients will need to bring their own blades [to the clinic] yes those sterile razor blades and at times the client won't even have a dollar to buy the sterile razor blade you understand. It's now the same as saying that the service is no longer free… Now they [clients] have to go an extra mile of being referred. They now have to incur transport costs or even go to the PSZ centres [non‐CHIEDZA] in the city or a specific place that they are referred to. (CHIEDZA provider‐IDI11, Female, Bulawayo, Phase 3)

According to CHIEDZA providers, outside CHIEDZA, youths experienced rationed, difficult, and expensive access to contraceptives. This preceded the national contraceptive shortage and COVID‐19. For many of these young women, these existing constraints were then made more acute by the pandemic.

### Pre‐COVID 19: CHIEDZA Perceived as a Source of Reliable, Acceptable, and Free Contraception

When the national contraceptive shortage occurred in October 2019, young women came to CHIEDZA in part because at pharmacies and clinics, these commodities, if available, were expensive.

What made me come to CHIEDZA to seek family planning services was the issue of the pills themselves being sold at exorbitant prices these days whether at the pharmacy or clinic. So at times, you won't afford to buy the pills on a monthly basis… I came to CHIEDZA to access family planning services because they are given for free. (Youth‐IDI1, Harare, female, 24 years, Phase 2)

CHIEDZA was viewed differently from other health care services because contraceptives were free and services integrated. Furthermore, youth did not have to navigate negative attitudes, seek financial support for commodities, or admit their sexual activities or need for SRH services. These features encouraged engagement with CHIEDZA and the uptake of family planning services.

There isn't any other place where they [young women] can get those services for free, and it is sort of taboo anyway in our tradition for a young girl to ask from her mother money to buy family planning pills. It is like taboo but because CHIEDZA came with these free services, it has become easier to access them for free. (Youth‐IDI17, Mashonaland East, male, 24 years, Phase 2)

Unlike at local clinics, at CHIEDZA, youth felt they could freely, safely, and openly take contraceptives and condoms. They felt comfortable talking to providers about their SRH needs and lives without fear of being criticized for being sexually active. One client described experience at the local clinic where nurses would say:

‘yeeeee at your age what do you need family planning for?!’ in a judgmental tone, while there was also no privacy: one ‘might bump into their neighbors’. This young woman came to CHIEDZA instead of the clinic for family planning because, ‘CHIEDZA makes one feel comfortable as there are only youths present and privacy is maintained. The clinic is not youth‐friendly.’ (Youth‐IDI7, Bulawayo, female, 24 years, Phase 2)

### COVID‐19 and Its Mitigation Measures’ Effects on CHIEDZA

Due to COVID‐19, three key factors coincided with reducing youths’ access to family planning services at CHIEDZA.

### Deprioritization of Sexual and Reproductive Health Care

In the few weeks leading up to the level 5 lockdown, CHIEDZA providers attempted to prepare clients by encouraging them to come for supplies before the closure, including through the youth mobilizers. This was to avoid interruptions in contraception and other commodity supplies in the hope that the supplies would see them through the lockdown period. However, in those last days, other immediate economic concerns provoked by the prospect of lockdown meant that attending CHIEDZA was not perceived as a priority by many youths. For clients who ordinarily engaged with CHIEDZA, as economic stress increased, the concerns about their SRH were subsumed by more pressing needs, such as having enough food to prepare for and survive the lockdown.

One of them [CHIEDZA youth mobiliser] was almost assaulted by people in the communities with them saying that we know that there is CHIEDZA at the hall; can you please just leave us in peace in our homes because right now we are focusing on mealie‐meal [food] such that when we go on lockdown we will have something to eat. (CHIEDZA provider‐IDI1, Bulawayo, female, Phase 1)

The health providers noted that the shifting priorities reduced the number of clients accessing CHIEDZA services in the weeks prior to lockdown.

By the time we got to Cluster‐X as the last centre we visit on [a specific week day], the numbers were even much less. Those who were coming were usually ladies who were coming for their family planning, and they were saying that they need their supplies because when the lockdown starts they don't know what will happen. (CHIEDZA provider‐IDI5, Harare, Phase 1)

### Growing Fear of Contracting COVID‐19 Kept Youth Away from Care Settings

According to CHIEDZA providers, youth also started to fear coming to CHIEDZA because they associated all health care settings with high infection risks.

Our regular clients they would boldly tell us that they will come after this COVID thing has passed. And then the other time it happened that I sneezed in the booth and the client I was with actually said, “aah sister do you want to give me COVID? What's happening here, please refrain from coming near me.” (CHIEDZA provider‐IDI1, Bulawayo, Phase 1)

Similar to the youth, the CHIEDZA providers also became anxious and afraid of contracting COVID‐19, as they had to travel to and from work using public transport and provide health services to the youth who were still coming to CHIEDZA. The quality of service delivery became compromised.

And when we were interacting with the clients, I would talk to them but with fear, because I didn't know who had COVID or not amongst the clients so I would try and maintain a distance. Of course, it wouldn't be a meter or 2 meters apart distance because our booths are very small…I would talk with the clients with hesitance and having some small reservations that I shouldn't judge a client, maybe they don't have the virus or they have it but I honestly didn't know. (CHIEDZA provider‐IDI4, Harare, Phase 1)

I was worried at times even you know talking to the client. We also had this fear that you didn't need to be with the client for a longer time. So the issue was like even if the clients were coming, they wouldn't get all the information that we wanted to give them because the provider is also you know having all the fears of talking to someone and the fear of spreading COVID. (CHIEDZA provider‐IDI2, Harare, Phase 1)

### Closure of CHIEDZA and Travel Restrictions

CHIEDZA providers noted that in anticipation of CHIEDZA's closure, to prevent pregnancy, some women took readily accessible, available, and/or affordable contraceptives at CHIEDZA even if the method might not have been their ideal choice.

…some of the clients were even changing their family planning methods…they were changing from tablets to injectables which last for 3 months and not because of the side effects of the pill that they were taking, but because of fearing that we won't be able to get the family planning pill if we lockdown because right now we were giving the one month supply. (CHIEDZA provider‐IDI7, male, Harare, Phase 1)

When CHIEDZA was about to close, there was little time for providers to prepare for and address all of clients' family planning needs. Both provider and youth participants described how some youths stopped using contraceptives and condoms during the lockdown.

I only stopped during the first lockdown when CHIEDZA was closed and I didn't have enough supply. (Youth‐IDI2, Harare, female, 22 years, Phase 4)

I know there's one boy in [cluster‐X] who would come every week to get condoms and one day I asked him like you come every week, can I know like are you using them or are you selling them? And he told me that, look I have got 4 girlfriends and I have slept with all of them in the week that you guys are away so I need these condoms and I said okay, what happens when they get finished? And he was straight up in saying that when they finish, then I do unprotected sex. (CHIEDZA provider‐IDI4, Harare, Phase 1)

Providers and youth agreed that closing down a readily accessible and youth‐friendly HIV and SRH intervention such as CHIEDZA for even six weeks left youth clients vulnerable to SRH problems.

The next big thing was family planning because the ladies we see at the centres always talk about how they appreciate the services and that they can get it for free and at the pharmacies, it's so expensive now so out there on the streets it's the same. So I know that people are going to be affected like those who have reached their resupply dates for family planning and the control pills. I know they will be affected by this because people don't have money to buy (them). (CHIEDZA provider‐IDI4, Harare, Phase 1)

During the lockdown, I failed to access my Depo because of the restrictions…I did not change my family planning, and I did not use anything. I am just lucky that I never fell pregnant, but I was not using any contraceptive. I was always in fear and scared. Men do not understand that you are not on contraceptives because all they want is sex, regardless of whether the contraceptive is effective or not that is none of his business to him. (Youth‐IDI13, Bulawayo, female, 24 years, Phase 4)

Unlike family planning and HIV testing, HIV care and treatment were considered essential services. Where feasible, youth living with HIV were supported and provided with three to six months of treatment to accommodate the lockdown and its restrictions.

For our CHIEDZA clients who are in the HIV cohort, there were arrangements that were made that they are all contacted to come and collect their resupplies and meet up with the nurses being given 3–6 months’ resupplies. So for those, I am not worried. (CHIEDZA provider‐IDI1, Bulawayo, Phase 1)

### Impeded Access to Safe Family Planning Services Without CHIEDZA

New challenges brought about by COVID‐19 reinforced existing ones, such as poverty and commodity stock‐outs, and some existing weaknesses in the health system appeared more pronounced. This affected family planning service use patterns. Three of these patterns are described here.

### Limited Alternatives to Accessing Contraceptives

In the absence of the readily available and accessible CHIEDZA services at a time of restrictions that minimized movement, some young women found alternative strategies to obtain contraceptives. Some of them or their partners risked harassment from the police by breaking travel restrictions and trying to find oral contraceptives at the few formal health facilities that remained open, while others told providers they bought contraceptives on the black market.

I stay close to the [neighborhood] so that's where my husband would go and buy from the pharmacy…. (Youth‐IDI9, Mashonaland East, 24 years, female, Phase 4)

After reopening CHIEDZA we were asking clients what they were doing to access family planning services during the lockdown and they will tell you that they bought some from [suburb‐black market] or in their communities there are some people who sell pills illegally. So we encourage them that they shouldn't buy pills from the illegal market because you won't know the expiry date of the pills or where they got them from. Because a lot of people sell painkillers like Ibuprofen and the family planning pills as well, for them to know where they come from is a question left unanswered…. (CHIEDZA provider‐IDI4, Female, Mashonaland East, Phase 3)

In Zimbabwe, the sale and purchase of contraceptives on the informal market occurred before COVID‐19, but lockdown restrictions likely increased it, as many young women had no other choice. Some young women were cognisant of risks associated with the black market, such as procuring expired oral contraceptives.

I realized through my interaction with clients that if you deny them the services they will find other ways to get the service, like they end up buying from those people selling the pills in the community. I then ask them does the person selling explain how to use the pills, check their vitals, and their compatibility with the pill. They will tell you that when they go to the clinic, the nurses there refuse to give them the pills so they end up buying them on the streets. (CHIEDZA provider‐IDI15, Female, Bulawayo, Phase 3)

For those who sell family planning pills at the tuck shops; it is scary to buy from them because they may be selling expired pills (Youth‐IDI9, Mashonaland East, female, 24 years, Phase 4)

Presented with limited alternatives, young women had to assess and weigh relative risks between the high likelihood of unintended pregnancies and accessing contraceptives by any means.

### Reduced (or Unreliable) Supplies by Trading in Contraceptives and Condoms

The outbreak of the pandemic did not diminish young women's contraceptive needs and exacerbated the hard socioeconomic conditions in Zimbabwe. The youth participants supported provider reflections on how the pandemic reduced women's ability to afford contraception.

Aaah, I remember [referring to 2020's lockdown] others used to buy them from the community health workers and some would get the pills in exchange for hard labor like doing laundry, slashing or cutting grass you know so that they would get money like $50 Bond to buy the family planning pills with…Other women couldn't do anything [no more hard labor opportunities] and I know quite a few who are pregnant now. (Youth‐IDI2, Harare, female, 22 years old, Phase 4)

In both the formal and black markets, contraceptives had to be purchased and were too expensive for many young women. Not only did youth have less money during the lockdown, but they had become accustomed to free supplies offered by CHIEDZA. According to youth participants, among those who could not readily afford contraceptives, some “borrowed” oral contraceptives from neighbors or peers who had a surplus.

You would hear people going around to their neighbors asking them if they have any extra supplies of family planning. (Youth‐IDI13, Harare, male, 25 years, Phase 2)

My mother is the one who gave me the batch to use up until I came back to CHIEDZA. She got them from a friend of hers who had bought extras, and she replaced them after buying her supply. (Youth‐IDI24, Mashonaland East, female, 18 years old, Phase 2)

When all other options were not possible, some young women discontinued taking contraceptives.

Recently, when we were still in total lockdown [level 5], I stopped taking them when my supply ran out and at the pharmacies. They were going for USD$1 for one pack and CHIEDZA was closed. (Youth‐IDI16, Harare, female, 24 years, Phase 2)

### Staying Away from “Unfriendly” Clinics

Having gained confidence in accessing the more reliable, supportive, and youth‐friendly services at CHIEDZA, youth were forced to reconsider seeking family planning services from the public sector facilities that remained open when CHIEDZA closed. In some instances, this compromised condom and contraceptive refills due to the fear of negative staff attitudes.

I stopped using condoms, and I feared going to the clinic because of what my friends said about how the nurses treated them. Plus, since we were on lockdown I feared the police as we were not allowed to move around. (Youth‐IDI4, Bulawayo, male, 19 years, Phase 2)

Public sector clinics were meant to remain open during the lockdown, as they were classified as providing essential services. However, according to the mobilizers, young women constantly asked them if and when CHIEDZA would reopen so that they could refill their contraceptives as they had run out. This implies that these young women may not have returned to the public sector clinics, could not afford to, preferred to avoid them, or the public sector clinics did not view family planning as an essential service.

### Challenges to CHIEDZA Access After Reopening

Once CHIEDZA reopened, mobility restrictions continued to constrain youth's ability to get to CHIEDZA. Some youth reported military or police resistance and harassment. From nonparticipant observations in the field and provider reports during team meetings, the police and military presence in the streets instilled fear of movement among young people. They were questioned and intimidated by the police as they were making their way to the CHIEDZA community centers. This interrupted access to CHIEDZA.

### Accessing Contraceptives on Behalf of Parents or to Resell

While reopening CHIEDZA reinstated youth's access to free contraceptives, according to the providers, it also put pressure on some of them to use CHIEDZA as a source of contraception for others (ineligible parents or siblings) or to convert it into a resource to generate some income (selling the free contraceptives on the informal market).

We discovered that some of these girls were getting these pills for resale. So some of them are in a bad space financially, and they do not have breadwinners to provide for them, so they then decide to come to CHIEDZA to get the pills and resell them to other people in their neighborhood…. At clinics, they screen people at the gate, so those who want condoms are turned away because collecting condoms is deemed not essential. Clients then end up coming to us to get condoms, possibly for resale. (CHIEDZA provider‐IDI15, Bulawayo, Female, Phase 3)

The only problem is that some mothers in the communities are now sending their 16‐year olds to CHIEDZA to take the family planning services on their behalf since they don't fit in terms of criteria of inclusion. So you usually catch out that they are lying when you start asking them questions like how they take the pills, what the pills look like or when they started taking the pills… They will eventually tell you the truth that they were sent by their mother to take the family planning pills on their behalf. (CHIEZA provider‐IDI4, Mashonaland East, Female, Phase 3)

In retrospect, health providers felt that CHIEDZA should have reverted to providing three months of oral contraceptive supply (as was done for ART) before the pandemic to reduce repeat visits to CHIEDZA and to subsequently ensure young women had sufficient supply during the lockdown. However, this could have increased the frequency of reselling or giving contraceptives to ineligible family members. CHIEDZA eventually decided to absorb this risk, and since mid‐January 2021, it has resumed providing a three‐month supply of oral contraceptives as part of COVID‐19 mitigation measures.

## DISCUSSION

This study highlights how COVID‐19 exacerbated existing barriers to youths’ ability to safeguard their sexual and reproductive health. Even before the COVID‐19 pandemic, youth faced considerable barriers to accessing HIV and SRH services. CHIEDZA was configured to address some of these barriers and improve access to and coverage of HIV and SRH services. Contraceptive demand by young women continued to be high during the pandemic, but their ability to ensure the continuity of their supply was threatened by the lockdowns, mobility restrictions and limited availability of youth‐friendly service options. When a service such as CHIEDZA, which is perceived as youth‐friendly, was disrupted by the pandemic, youth sought alternative, often suboptimal, pathways for accessing and using contraceptives during the pandemic. Some stopped engaging with any care system and went without contraceptives as they waited for CHIEDZA to reopen.

CHIEDZA is based on empowering youth to act with autonomy by providing direct (and free) health services. During closure, some young women had to procure contraceptives at public clinics, private pharmacies, or the black market, all of which incurred costs. Respondents underscored the ways that crisis‐related barriers to using family planning services were compounded by preexisting poverty. However, reopening CHIEDZA illustrated uneven access disaggregated by age. Youth who are often considered the most underserved had a greater degree of protection through CHIEDZA than their adult siblings and mothers, as their needs were generally considered to be better met. Thus, the pandemic revealed existing gaps in family planning access.

Research on the accessibility of SRH services for youth shows that there are many different components, including availability, affordability, acceptability, and quality services (Mazur, Brindis, and Decker 2018; WHO 2011; Denno, Hoopes, and Chandra‐Mouli 2015; Ndayishimiye et al. 2020). This study showed that youth will prioritize different components, and when options become limited, compromises on these priorities will be made. Prior to the COVID‐19 pandemic, due in part to existing difficult socioeconomic conditions, CHIEDZA offered affordability and acceptability. When the intervention was closed temporarily, acceptability was compromised for some as they engaged with less ideal care settings, and some youth pursued the only affordable and available options. Additionally, as the pandemic persisted and CHIEDZA reopened with COVID‐19 control measures in place, the perceptions about CHIEDZA that made it acceptable shifted with the pandemic. CHIEDZA was originally designed in response to what youth had said they wanted: a place where they can access youth‐friendly health services that do not look or feel like a health facility. However, with the advent of the COVID‐19 pandemic, CHIEDZA's status as a health care setting became more visible, both because the service sought recognition as an essential service provider and because growing fears of COVID‐19 led to it being associated with potential risk of infection.

This study demonstrated the underinvestment and low attention to family planning for youth when compared to other SRH services such as HIV. In Zimbabwe, at the advent of the COVID‐19 pandemic, within the health system, every effort was made to ensure effective continuity of care for HIV so that people living with HIV had adequate ART supplies to weather the lockdowns (Ministry of Health and Child Care 2020). In contrast, there was little attention invested in family planning, despite recognized adverse short‐ and long‐term consequences of unintended pregnancies. Thus, we found that some young people switched contraceptive methods prelockdown or borrowed oral contraceptives during the lockdown. Although this demonstrates their willingness to adapt and make active decisions in the context of a fragile system, it also shows a lack of prioritization of family planning services despite government pledges to reduce unmet needs.

A substantial body of research has focused on health systems preparedness and resilience during crises (Kruk et al. 2015; Nuzzo et al. 2019; Kieny and Dovlo 2015; Martineau 2016; Fridell et al. 2020). Resilience became particularly relevant during the COVID‐19 pandemic (Kieny and Dovlo 2015). Resilient health systems can operationalize a robust public health response during a crisis and provide an effective service delivery system during noncrisis times (Kruk et al. 2015; Martineau 2016). Such a health system must be able to maintain its core functions when a crisis occurs (Sundararaman, Muraleedharan, and Ranjan 2021; Doubova et al. 2021). Zimbabwe's health system was already fragile, with core functions compromised even before the COVID‐19 pandemic (Meldrum 2008; Makoni 2020; Green 2018; Kidia 2018). Therefore, the system's capacity was rapidly overwhelmed. Our study reflected conditions in peri‐urban and urban communities and systems in Zimbabwe. Predictions and simulations were made about disrupted access to SRH in other parts of Africa (Govender, Naidoo, and Taylor 2020; Sen and Govender 2015) and other parts of Zimbabwe (Murewanhema 2020). Decisions were made to suspend mobile outreach provider teams, shrink geographical coverage, and limit service provision at static clinics; in some instances, the provision of LARCS was terminated (Church, Gassner, and Elliott 2020). This indicates that it is likely that young women in remote and rural settings experienced harsher disruptions and interruptions to contraceptive use and access to care due to COVID‐19. Discussions on COVID‐19 recovery strategies have included building health systems resilience and preparedness (Kruk et al. 2015; Sundararaman, Muraleedharan, and Ranjan 2021; El Bcheraoui et al. 2020). Crises such as the COVID‐19 pandemic present learning and systems transformation opportunities so that health systems do not return to the original vulnerable state but rather become continuously adaptive systems that can withstand shocks (Fridell et al. 2020). For example, the presence of protected conditions such as HIV care and treatment during a pandemic shows that, with attention and investment, it is possible to have robust systems in place for other conditions such as family planning, which can weather crises.

Since the COVID‐19 pandemic began, commentaries (Hussein 2020; Aly et al. 2020; Kumar, Malviya, and Sharma 2020; Hall et al. 2020) and empirical studies on the impacts of COVID‐19 on SRH have emerged (Gilbert et al. 2021; Bolarinwa 2021; Lewis et al. 2021a; Both, Castle, and Hensen 2021; Yarger et al. 2021; Endler et al. 2021; Balachandren et al. 2022; Steiner et al. 2021). Some studies, such as ours, found that access to contraception became difficult during lockdowns (Balachandren et al. 2022), and others focused specifically on the impact on adolescents and young people (Lewis et al. 2021a; Both, Castle, and Hensen 2021; Yarger et al. 2021; Steiner et al. 2021). Two of these studies have shown findings that support our findings (Both, Castle, and Hensen 2021; Lewis et al. 2021b). In Scotland, an online survey was used to investigate the effects of COVID‐19 on condom and contraceptive access and use among 16‐ to 24‐year olds. Disrupted prevention care included unanticipated contraceptive pathways and switching from freely provided to commercially sold contraceptives to mitigate disrupted access (Lewis et al. 2021b). A mixed‐methods study including 2700 youth from six low‐ and middle‐income countries, including Zimbabwe, found that 30 percent of young women in the survey were unable to access the contraceptives they needed due to fear of catching COVID‐19 in health facilities, lack of transport, and closure of health facilities (Both, Castle, and Hensen 2021). Different contraceptive pathways, purchasing contraceptives and fear of catching COVID‐19 were effects that were also present in our study. Unlike in Scotland, where switching to purchasing contraceptives was affordable, affordability was limited for the young people in our study, as contraceptive prices were inflated due to rationed supply.

Studies focusing specifically on the impacts of COVID‐19 on SRH in LMICs are scarce (Mukherjee et al. 2021), and those on young people's SRH in LMICs remain limited (Seme et al. 2021; Peters et al. 2021; Meherali et al. 2021). Our study adds to this body of knowledge and reflects how SRH is often sidelined and forgotten during crises.

The study had limitations. It was conducted during the pandemic period when perceptions and knowledge of COVID‐19 were constantly evolving. This could affect retrospective data interpretation, as interviewees’ knowledge and perceptions of COVID‐19 may have shifted depending on when they were interviewed. The temporal specificity of the data is not unusual, but it becomes potentially more explicit as our developing understanding and knowledge of COVID‐19 may illuminate how the existing empirical context shaped interpretations and experiences. The findings are also restricted to the context of the CHIEDZA trial, and therefore, some of them cannot be generalized in the standard of care. However, the lessons that can be absorbed into the standard of care and health systems are illustrated in this section. The CHIEDZA trial has responded as quickly as it can to adapt to the new challenges triggered by the pandemic. Ongoing research will capture to what extent service engagement has and can be maintained and to what extent there have been losses in satisfaction (if any), which could be detrimental to contraceptive uptake.

## CONCLUSION

Although youth used alternative settings of care to access contraceptives during the COVID‐19 pandemic, our study demonstrates that the sustained provision of quality youth‐friendly services is needed for access and use of contraceptives, even during crises. The COVID‐19 pandemic revealed existing systemic and structural gaps in SRH service provision. They were made worse by the pandemic and underscores the importance of maintaining access to broader health services as part of epidemic readiness and preparedness.

## CONFLICTS OF INTEREST

The authors declare no conflicts of interest.

### AUTHOR CONTRIBUTIONS

Constancia V. Mavodza, Joanna Busza, and Sarah Bernays conceptualized the study. Constancia V. Mavodza, Rangarirayi Nyamwanza, and Portia Nzombe collected the data. Constancia V. Mavodza analyzed the qualitative data with support from Joanna Busza, Sarah Bernays, and Constance R.S. Mackworth‐Young and then wrote the first draft. Ethel Dauya, Chido Dziva Chikwari, Mandikudza Tembo, Constancia V. Mavodza, and Rashida Abbas Ferrand implemented the CHIEDZA trial. Katharina Kranzer participated in the COVID‐19 adaptations of the trial. Rashida Abbas Ferrand is the PI of the CHIEDZA trial. All authors provided input to the draft manuscript and read and approved the final manuscript.

## FUNDING

This work was supported by funding from the Wellcome Trust under Grant Number: 206316_Z_17_Z and the Fogarty International Center of the National Institutes of Health under Grant Number: D43 TW009539.

## Supporting information

Supplementary material

Supplementary material

Supplementary material

Supplementary material
