# Supplementary material for: Interrupted Access to and Use of Family Planning Among Youth in a Community‐Based Service in Zimbabwe During the First Year of the COVID‐19 Pandemic
Source: Stud Fam Plann. 2022 Jun 22:10.1111/sifp.12203. Online ahead of print. doi: 10.1111/sifp.12203 (PMC9350188; doi:10.1111/sifp.12203)
Supplement: Supplementary file 1 — Supplementary material [file SIFP-9999-0-s004.docx]

**Tools for CHIEDZA Process Evaluation on COVID-19**

**Interviews with CHIEDZA staff**

**Interviews with CHIEDZA staff**

1. What have you heard about Coronavirus? What do you understand about it?
   1. How knowledgeable do you feel you are about COVID? What are your information sources.?
2. How are you feeling personally about the Coronavirus situation?
   1. How has your life changed so far in response / preparation for Coronavirus?
3. How has your work in CHIEDZA changed so far in response / preparation for Coronavirus?
   1. Do you have any concerns with how CHIEDZA happened during COVID? How much support have you been receiving- was it adequate? What kind of support do you think you would have wanted or still want?
   2. based on your experiences/talks with CHIEDZA stakeholders, what about the CHIEDZA communities? The clients? How have they been responding to COVID?
4. As a healthcare worker (in CHIEDZA or otherwise), what are your views on the COVID situation?
   1. do you think the measures are adequate? What would you want to see more of?
   2. Based on your experiences, how feasible do you think changes (the lock down) might be in Zimbabwe?
   3. Do you think people will comply with the lockdown? or other COVID interventions?
5. Can you tell me about other changes that you know of, outside of your own life and work, that are being made in response or preparation for Coronavirus?
6. What changes do you think could be made to help and support healthcare workers?
7. What do you think the general public’s perception of Coronavirus is? What is most people’s understanding around it? Where to most people get their information around it?
   1. CHIEDZA clients? Have they talked to you about it? What has been the general feeling?
   2. How did doing CHIEDZA start to change because of COVID? based on interventions?
8. What do you think the health impact of Coronavirus might be? What impact do you think COVID; and the lockdown would have- on health services in general/ What about on CHIEDZA services? (HIV, Family Planning, MHM, might have?)
   1. Do you think there might be health impact of Coronavirus beyond the disease itself?
9. Who do you think is the most vulnerable because of COVID? why? or would suffer the worst of the pandemic.
10. How does the Coronavirus situation compare to your experience of other disease outbreaks or times of widespread upheaval?
11. What would you like to know about Coronavirus if you could?
