# Supplementary material for: Interrupted Access to and Use of Family Planning Among Youth in a Community‐Based Service in Zimbabwe During the First Year of the COVID‐19 Pandemic
Source: Stud Fam Plann. 2022 Jun 22:10.1111/sifp.12203. Online ahead of print. doi: 10.1111/sifp.12203 (PMC9350188; doi:10.1111/sifp.12203)
Supplement: Supplementary file 2 — Supplementary material [file SIFP-9999-0-s001.docx]

**IDI Topic Guide for CHIEDZA youth champions/ mobilisers**

**INSTRUCTIONS for the Interviewer: How to use the IDI Guide**

***The ultimate goal of the data collection is to inform: implementation, mechanisms of change and context of the CHIEDZA intervention in light of the COVID-19 pandemic***

1. There are two levels of questions:
   - Primary interview questions: appear in **bold** text. They address the topics that you as the interviewer must ask and discuss with participants. The questions are suggestions for getting the discussion going. You are not required to read them verbatim, but they are written to ensure some consistency across IDIs. They are not exhaustive and they are not prescriptive. This means that as the interview progresses, you may ask questions that are not included below, and similarly, it may not be appropriate or necessary to ask all of the questions included in this topic guide- *the discussion should be guided by what your participant says, NOT by the topic guide.* For that to happen, you should make sure that you’re familiar with the guide so that so that you can engage more fully in the discussion, and be responsive to what the participant is telling you, by exploring these responses further. Try to integrate some of the information that they have told you into your subsequent questions – this will demonstrate that you’re listening, and give participants a chance to clarify anything you might be misunderstanding. It is important for you to show that you are interested in what they are saying, and that you are there to learn from them.
   - Probing topics: are indicated with a bullet. These are to assist and encourage further discussion with a participant who may be providing very little information. It's not a requirement to cover every probe. Which probes you may or may not ask will depend on what has already been discussed.
2. Words found in (parentheses) are meant to tag the memory of the interviewer in relation to PE objectives.
3. The IDI guide is divided into two columns.
   - **The left-hand column** contains the research questions and probes to be used during the IDI with CHIEDZA clients.
   - **The right-hand column** contains the research questions and probes to be used during the IDI's with non- CHIEDZA clients
4. The interviewer (with permission from the participant) should take notes and these notes should be labelled with initials, participant's ID, IDI guide is not meant to be used to take notes. Rather, a separate notes form, where with interviewer initials, the participant’s PTID, as well as the date, start and end time of the interview should be used.

**Before starting the IDI, ensure the participant has provided written informed consent**

**Interviewer:**

**Date: ..........................................................................**

**Interview Start Time: .......................................**

**Interview Stop Time: .......................................**

**Participant ID (initials/cluster letter/XX): ..............**

**Pre-interview background questions**

Thank you for giving your consent to be interviewed. Before we begin it would be really useful to collect some background information about you and I assure you that the information you provide will be kept strictly confidential and anonymous.

**We will begin with completing some Demographic Information below. Please let me know if you have any questions completing this**

| **1. Age group (years):** |  | | |
| --- | --- | --- | --- |
| **2. Cluster:** |  | | |
| **3. Gender (tick):** | Male | Female | Other |
| **4. Are you currently in a relationship? (tick)** | Yes | No | decline to answer |
| **5. Marital Status (tick):** | Married | Single | Divorced |
|  | Widowed | Separated | Unmarried (living with partner) |
| **6. Residential status (tick)** | With partner | With Parents | On own |
| **7. Are you sexually active** | Yes | No | decline to answer |
| **7b. If yes, how many sexual partners:** |  | | |
| **7. Education level achieved (tick)** | Primary | Secondary | University/College |
|  | None |  |  |

**[*Read Introduction*]**: Thank you for taking the time to speak with me today.

My name is _______________ and I am working with the CHIEDZA research project to learn more about sexual and reproductive health and HIV testing and treatment services for adolescents and young people aged 16-24 in Zimbabwe.

I would like to talk to you about your own experiences, ideas and opinions regarding COVID-19, HIV and sexual and reproductive health for adolescents and young people at CHIEDZA, in facilities and within the community that you live. Specifically, I would like to learn about your experiences accessing health services, and what kinds of services you have/are using. I am also interested in learning about the challenges that young people face trying to access health services, and how you think their access to, and use of these types of services can be improved. Additionally, I would like to also talk to you about your experience of Coronavirus, the lockdown and accessing health services during this time.

The interview will take about 30-45 minutes. I appreciate you spending this time with me.

I am going to audio record the interview to make sure that I capture all the valuable information that you share with me. I may also write things down while we’re talking so that I don’t forget anything. Participation is voluntary- you do not have to answer any question that you don’t want to, and you can choose to stop the interview at any time.

Everything you say is confidential, so please feel free to talk about your experiences and ideas. We will not record your name anywhere, and no one else will hear the tape or see the notes besides the people who are working on this research project. We may use some of what you say in reports or publications, but will never use your name.

If you have any questions about this study, you can ask me now, or at any time during our conversation ***(RA: make sure you have collected signed consent form and answered any questions.)***

Start the ***tape recorder***.

Before we start, can you confirm for the recorder that you have already provided written informed consent to take part in this discussion? [*Wait for oral confirmation to begin*].

Thanks for taking the time for this interview. As you know, CHIEDZA is reopening, and I wanted to ask you a few questions about your thoughts about CHIEDZA before it closed as well as reopening, and also your feelings about coronavirus. Feel free to stop me at any time, and also to add in anything that you want to share.

Thanks for taking the time for this interview. As you know, CHIEDZA is reopening, and I wanted to ask you a few questions about your thoughts about CHIEDZA before it closed as well as reopening, and also your feelings about coronavirus. Feel free to stop me at any time, and also to add in anything that you want to share

**COVID Experiences**

1. **To begin with, can you tell me how your life has changed over the last few weeks/since COVID-19 happened?**
   - Can you describe what has been going in your communities during this lockdown?
2. **How have you felt during the lockdown?**
3. **What are your views about coronavirus? about the lockdown?**
   - How do people in your community feel about coronavirus, and about the lockdown?

**CHIEDZA experiences**

1. **Can you tell me what you know about CHIEDZA? (*probe bulletpoints below*)**

- Please describe how or where you heard about CHIEDZA? What made you come here?
- How many times have you come to CHIEDZA? If more than once, how has your experience changed over time? What did you think the first time you came and has that changed over time?
- What services were you seeking when you came to CHIEDZA? Are there other additional services that you took up-why did you take them up? ( and when did you take them up?)
- Can you describe what happens when you come to CHIEDZA?
  - How long do you wait before seeing a health provider? and how do you feel about the wait time?
  - What about in the health booth: How are your interactions with the health providers? What are your views on how you are treated? How do this compare to other clinics or hospitals you have gone to before?

| **Family Planning Questions**  ***ask Q1 to every participant male & female. Q2 onwards is for female clients & those who take on a FP method)*** |
| --- |
| 1. Can you tell me what you know about FP?   - Contraceptives? Pregnancy prevention? |
| 2. Can you tell me about any family planning services you received at a facility or here at CHIEDZA?   - Can you describe what happened during that visit? - Reasons for seeking FP services? Had you come to CHIEDZA for FP services or something else? - Why CHIEDZA (and not some other Health facility for example)? What did you like about this service? Has this changed with time? |
| (For those who use a FP method):   - Please describe the contraceptive method you currently use? How do you feel about your Family planning method? When did you start using it and why did you decide to use a FP method? Is this from CHIEDZA or elsewhere? - Is this your choice of method? How did you decide on the method- alone, partner, parents? Is this method working for you? - Influence of FP method and service on 1) knowledge, 2) use, partner relationships - In your experience, how are you using/taking your contraceptive? *(adherence)*   - How are you able (or not) to use it as described by the CHIEDZA providers?   - are there any personal, community, religious, partner factors influencing adherence?   - How does CHIEDZA influence your ability to take contraceptives? - Tell me about a time you stopped your contraception.   - What was going on? Did you fix it, if so how? Did you seek assistance from anyone (CHIEDZA?)? |
| Since CHIEDZA, how has your knowledge or use of FP changed?   - worries about FP & reasons for worry - behaviour changes, contraceptive method changes, |

- What do you think has been working well in CHIEDZA? What do you think has not working well? Would you suggest doing anything differently?

**CHIEDZA and COVID Experiences**

1. **How have you found it while CHIEDZA has been closed? (probe bulletpoints below)**

- What are your views on CHIEDZA being closed for over a month due to COVID-19 and the lockdown?
- How where you accessing health services (HIV, FP, condoms, MHM, STI,) during the lockdown?
  - Can you tell me about any health services that you needed but could not access because of the lockdown/because CHIEDZA was closed?

1. **How do you feel about CHIEDZA reopening, at this time?**

- One of the justifications for reopening CHIEDZA is that it provides essential services for young people. What are your views about this?

**Of course, CHIEDZA is reopening but had to make some changes because of COVID. Some of parts of CHIEDZA may no longer be possible.**

1. **What do you think are the parts of CHIEDZA that should not be changed/moved if it's going to keep working/being accessible to young people?**

- What are the parts that you think even if we removed them, CHIEDZA would still be fine?
- In your opinion, what is the minimum that is needed for CHIEDZA to still be attractive to young people?

We have come to the end of the questions that I had for this interview. Do you have anything else you want to add or questions you would like to ask me?
