# Supplementary material for: Interrupted Access to and Use of Family Planning Among Youth in a Community‐Based Service in Zimbabwe During the First Year of the COVID‐19 Pandemic
Source: Stud Fam Plann. 2022 Jun 22:10.1111/sifp.12203. Online ahead of print. doi: 10.1111/sifp.12203 (PMC9350188; doi:10.1111/sifp.12203)
Supplement: Supplementary file 3 — Supplementary material [file SIFP-9999-0-s002.docx]

| We have four main areas that we would like to investigate in these interviews:   1. Topic 1: Providers’ experiences and perceptions of reopening CHIEDZA with the adaptations because of COVID-19 2. Topic 2: Broader experiences of being a provider in CHIEDZA 3. Topic 3: Experiences of providing family planning services 4. Topic 4: Experiences of providing STI services   The questions in this topic guide are not exhaustive and they are not prescriptive. This guide is to help you understand the primary areas of interest to pursue in the interviews. This guide should also give you some suggestions about how to word questions and approach the topics so that they may be likely to feel increasingly comfortable talking to you. They are just example questions. Ideally you should not take this guide into all your interviews, but make sure that you are familiar with it so that you can be responsive to what the participant is telling you through listening- but be confident that you are exploring the primary topics of interest. However, the most important point is to listen to what the young person is telling you and respond to that. Try to integrate pieces of information that they have told you into your questions at various points of the interview to demonstrate that you are concentrating and listening to what they are saying. You need to show that you have a compassionate curiosity to understand what it is that they are going through, what helps and what could be adapted to help them more. | | | |
| --- | --- | --- | --- |
| **Key areas of investigation** | **Rationale** | **Example questions** | **Explanatory notes** |
| **Topic 1: Experiences and perceptions of re-opening CHIEDZA** | | | |
| **How reopening CHIEDZA impacted them as providers?** | We would like to understand their personal experience of reopening CHIEDZA. As we’ve already spoken with clients around reopening CHIEDZA, it’s important to understand CHIEDZA reopening from the providers’ point of view. | How did you feel about CHIEDZA reopening?  How did you feel about the process of reopening?  Was there any part of the reopening that you found particularly challenging? | It’s important to recognise that they were interviewed during lockdown, and so shared their experiences and perceptions of CHIEDZA shutting down then. |
| **Their perceptions of how the intervention has changed** | We would like to understand their perceptions of how the intervention has changed now because of COVID-19. We would like to hear from them what they think the impact of these changes have been on the intervention from their perspective as providers. | How has CHIEDZA changed since it has reopened?  How have these changes impacted your work?  What are your perceptions of these changes?  How do you thing the changes have impacted clients’ interaction with and perceptions of CHIEDZA. | It would be good to recognise that the process evaluation team have been conducting observations in the CHIEDZA sites, and interviews with the mobilisers. The providers may have seen the team doing this data collection. So, it would be good to emphasise that we’re really looking for their views, as providers on how CHIEDZA has changed for them, and the impact this has had. |
| **Impact of change of CHIEDZA timings on providers’ workload** | The providers talked about how the change in timings of CHIEDZA (i.e. stopping at 3pm, rather than staying later in the afternoon/ evening), means that the clients are concentrated between 12-3pm. This leads to high workload and being very busy during this time. We want to find out more about this, in particular because this may produce a tension between quantity of clients vs. quality of care. Additionally, if the very long hours (including early picking up and late dropping off to their homes) impacts their motivation, this may impact the work they do, and how youth-friendly/ caring they are with clients. | Can you tell me about how the timings of CHIEDZA have changed?  How has this impacted your work as providers?  How do you feel about this change?  How does it influence how you deliver services?  How does it influence how you perceive CHIEDZA?  How do you think it has changed clients’ perception of CHIEDZA? | I think it would be good to really probe about this. How it impacts their work? How it makes them feel about their work? How it influences their motivation as providers? It would be good to really understand quite deeply the impact that this change has on the way they work, feel, and the sort of service provision they provide. |
| **Topic 2: Broader experiences of being a provider in CHIEDZA** | | | |
| **Their perceptions on CHIEDZA as an intervention** | We would like to understand how they perceive CHIEDZA, and what within the intervention works well, or doesn’t work so well. | What do you think about the CHIEDZA intervention as a whole?  How accessible do you think it is for young people?  What do you think about the package of services provided through CHIEDZA?  What challenges do you think the intervention holds?  How do you think CHIEDZA is different from services in health facilities?  How do you think CHIEDZA has changed since it first started? | It may be difficult for them to think about CHIEDZA outside of the changes because of COVID-19. So, it might be useful to as providers to think of a particular day before changes because of COVID-19, and ask them to describe their experience then. |
| **How they feel about their work as providers for CHIEDZA** | We would like to understand how they feel about their role and their work as providers within CHIEDZA. This is more to understand how it impact themselves and their life. | Can you describe your role in CHIEDZA?  How do you find working as a provider for CHIEDZA?  (nurses/CHWs) Describe what happens when a client comes to CHIEDZA and into your health booth (a consult).  What challenges do you encounter within your work in CHIEDZA?  Are you able to provide all the services or products that clients need? If not, why? How do you handle this situation?  How do you feel your work affects you and your life?  Do you have any particular stories of your experience as a provider in CHIEDZA that you would like to share?  Are there any particular experiences with a clients that you would like to share? | Here I think we really want to understand their personal experience and interaction with CHIEDZA. It would be good to encourage them to share personal and sensitive stories. |
| **Topic 3: Experiences of providing family planning services** | | | |
| **Knowledge of Family Planning** | Part of the reason why there is low uptake of LARCS (nationally) for example, is that providers do not have enough information/knowledge about them to impart to clients. We would like to understand CHIEDZA providers' knowledge of Family Planning and how this knowledge is used in providing FP services to young people in CHIEDZA | Tell me what you know about FP?  Describe any kind of FP training you have received  Aspects of personal life, community, religion, culture (context)  Since CHIEDZA, how has your knowledge or provision of FP changed?   - worries about FP & reasons for worry - behaviour changes, contraceptive method changes |  |
| **Service Provision Experience ( Acceptability)** | We would like to understand the Family Planning service provision story from the provider's perspectives.  We want to understand how they are implementing family planning services in this integrated environment, as well as understanding whether this implementation model is acceptable to them. | Describe your experience of providing FP services CHIEDZA  Describe what occurs when a young female client comes into the health booth seeking FP health services.  From your experience, what is the most common FP issue/product that young women seek in CHIEDZA? Why do you think this is?  Are you able to provide all the FP services or products that clients need? If not, why? How do you handle this situation? ( further probe about Termination of pregnancy services)  What approaches, if any, have you used to ensure young people get FP information/ services at CHIEDZA  What challenges do you face in providing FP services?  What sorts of questions or concerns do young women raise about FP the health booth? Are you able to address these questions or concerns? (provide an example if possible?) |  |
| **Access to Family Planning** | We would like to understand what providers perceive are the main issues in access to family planning, | In your opinion, what can be done to improve access to FP services for young people?  In CHIEDZA, what can be done? What about generally?  What do you think are the main issue affecting young people's access to SRH services? Are there any gaps? The opportunities?  In your opinion, what challenges do young people face in accessing FP services? What about challenge in uptake and use of contraceptives?  In your opinion, are there any services that young people need in particular? Are any of these not being provided by CHIEDZA? What do you think that young people need to have in place in a service to want to access them?  (further probe around Termination of Pregnancy services) |  |
| **Contextual Influence** | We want to understand, from the provider' perspectives, the context in which Family planning services are being provided | Can you describe any issues that have influenced your ability to provide FP services/products; or influenced clients ability to access and take up FP services/products   - the national contraceptive shortage started in August 2019: how has CHIEDZA and CHIEDZA clients been affected by this? (give examples if possible) - The doctors' & nurses' strike? - Laws, policies, religion, culture? - Partners/stakeholders in the community (give examples if possible) - client relationships/personal situations (give examples if possible) |  |
| **Topic 4: Experiences of providing STI services** | | | |
| **Reflections on offering STI services in the STI pilot last year** | STI screening was piloted last year within CHIEDZA and is going to be re-introduced in September/ October this year. We would like to learn from providers’ experiences of offering STI screening last year, and understand their perceptions on providing this service last year. | What was your experience of offering STI screening in the pilot last year?  How was STI screening integrated into the other CHIEDZA services?  What challenges did you encounter with STI screening?  How did you overcome these challenges?  Testing uptake increased over the course of the pilot. How was that achieved?  How do you think clients viewed STI screening?  Do you have any particular stories or experiences of offering STI screening that you would like to share? | Here it would be good to get at both the operational side of the service, as well as their personal experiences as providers. |
| **Reflections on offering STI testing for symptomatic clients in CHIEDZA** | Currently, in CHIEDZA (outside of the pilot last year) STI testing is offered, but only for clients with symptoms (syndromic management). We would like to understand their experiences of providing this service. | How does offering STI testing only for those with symptoms differ from the STI screening?  From your perspective, how have you found offering STI testing for symptomatic clients?  How do you think clients perceive this service? |  |
| **Suggestions for the re-introduction of STI screening in CHIEZA** | We would like to garner their ideas and suggestions of ways to improve STI services within CHIEDZA, in order to help us design STI screening services when they will be added to the CHIEDZA package of care. | What improvements would you suggest for better provision of STI screening within CHIEDZA?  How do you think uptake of testing could be improved?  How do you think linkage to treatment could be improved?  How do you think the partner notification process could be improved? | We need to recognise here that they may not have all the answers of having solutions to improve the STI screening services. |
| **Recommendations for feasibility, scalability, sustainability** |  | In your opinion, what could be done to make CHIEDZA better? FP/SRH services better |  |
| **Topic 5: Experiences of using on-site GeneXpert testing at CHIEDZA sites (Bulawayo providers only)** | | | |
| **Use of the GeneXpert machine and impact on role** | In Harare and Mashonaland East, the GeneXpert machine for STI testing was in a central lab. However, at CHIEDZA sites in Bulawayo, the GeneXpert machine was situated on-site in the community centres. CHIEDZA providers managed the GeneXpert machine, allowing for same day results for some clients. We would like to learn from providers’ experiences in using the GeneXpert machine and incorporating it into their role at CHIEDZA. | Did your role change after STI screening was introduced at CHIEDZA?  What proportion of your time at CHIEDZA was spent using the GeneXpert machine?  How was your experience doing STI testing using the GeneXpert machine?  Were there any aspects of using the GeneXpert machine that you found difficult?  Did you feel adequately trained to use the GeneXpert machine?  Are there any aspects that you would have liked to have received more training or support on? |  |
| **Reflections on the effect of using the GeneXpert machine on workload and flow** | Providing on-site STI testing in community settings is currently a very unique situation in Zimbabwe. The experience in Bulawayo may help us learn how to incorporate point-of-care tests for STIs into healthcare services more generally as they become more widely available. We therefore want to understand from the providers’ perspective the effect of using the GeneXpert machine on both workload and client flow. | How did incorporating GeneXpert testing affect your workload?  How did it affect waiting times and flow of clients through CHIEDZA?  What did you think about the space available to provide GeneXpert testing at the CHIEDZA sites?  Has providing same day results for STI testing been successful at CHIEDZA? |  |
| **Perceptions on the effect of using the GeneXpert machine on clients** | Potentially having same day results is also likely to be a novel experience for clients attending CHIEDZA. We would therefore like to explore how the providers perceive the effect of on-site GeneXpert testing on clients. | Did many clients receive their results on the same day?  Did many clients wait for their results?  What did clients think about getting same day STI results at CHIEDZA?  Why do you think clients chose to wait for their STI results?  Why do you think clients chose not to wait for their STI results? |  |
| **Thoughts of the providers on how the provision of STI testing at CHIEDZA sites in Bulawayo differs from that in Harare or Mashonaland East** | As on-site testing is not provided in Harare or Mashonaland East but is provided in Bulawayo, we would like to hear the providers’ opinions on if they feel provision of on-site testing has been a positive or negative experience. | Do you feel that providing results on the same day as testing is advantageous?  How does it make you feel that same day STI results can be provided in Bulawayo but not at CHIEDZA sites in Harare or Mashonaland East?  What are the main challenges to providing on-site STI testing at CHIEDZA?  Would you change how STI testing has been implemented at CHIEDZA sites in Bulawayo? |  |

The interviewer should give space for the interviewee to add any further details that they want to add, or ask any questions.

The interviewer should say that we’ve come to the end of the interview. They should thank the interviewee for their willingness to talk and participate in this discussion. They should explain how the interview will be used, and reiterate that information that the interviewee has shared will inform CHIEDZA and wider research, and things they have said may be quoted, but that it will not be linked back to the particular interviewee.
