# Supplementary material for: Interrupted Access to and Use of Family Planning Among Youth in a Community‐Based Service in Zimbabwe During the First Year of the COVID‐19 Pandemic
Source: Stud Fam Plann. 2022 Jun 22:10.1111/sifp.12203. Online ahead of print. doi: 10.1111/sifp.12203 (PMC9350188; doi:10.1111/sifp.12203)
Supplement: Supplementary file 4 — Supplementary material [file SIFP-9999-0-s003.docx]

| We have four main areas that we would like to investigate in these interviews:   1. Topic 1: Young women's knowledge, experiences and perceptions about contraceptive side effects, myths and misconceptions 2. Topic 2: Experiences of youth-friendliness in CHIEDZA in relation to Family planning service delivery 3. Topic 3: COVID-19 effects on family planning/contraceptive experiences 4. Topic 4: Young women living with HIV's experiences and use of family planning services in CHIEDZA   The questions in this topic guide are not exhaustive and they are not prescriptive. This guide is to help you understand the primary areas of interest to pursue in the interviews. This guide should also give you some suggestions about how to word questions and approach the topics so that they may be likely to feel increasingly comfortable talking to you. They are just example questions. Ideally you should not take this guide into all your interviews, but make sure that you are familiar with it so that you can be responsive to what the participant is telling you through listening- but be confident that you are exploring the primary topics of interest. However, the most important point is to listen to what the young person is telling you and respond to that. Try to integrate pieces of information that they have told you into your questions at various points of the interview to demonstrate that you are concentrating and listening to what they are saying. You need to show that you have a compassionate curiosity to understand what it is that they are going through, what helps and what could be adapted to help them more.  **Initially, Try to conduct these interviews entirely narratively and open-ended, without any topic guide. experiment with an entirely conversational method, where you go into the interview and know you want to address a) norms around young women & contraception b) determinants and experiences of contraceptive use c) experiences of CHIEDZA and d) changes due to COVID-19, both in needing and using contraception.** | | | |
| --- | --- | --- | --- |
| **Key areas of investigation** | **Rationale** | **Example questions** | **Explanatory notes** |
| **Topic 1: conversation about existing and previous sexual relationships and young women’s thoughts about becoming pregnant (and perhaps experiences of doing so), and how the young woman has developed her thoughts about when it is good to have a pregnancy (and who should, in what circumstances and how she will know if she is “ready”) vs when it is undesirable to have a pregnancy (situation, age, relationship)** | | | |
| **Young women's knowledge, experiences and perceptions about becoming pregnant (or not), and the use (or non-use) of contraceptives,** | We want to explore what young women think and know about methods/ ways of preventing pregnancies (including contraception) in different kinds of situations and also understand about what role partners/relationships play in wanting/ not wanting pregnancies.  We also want to explore where they get this knowledge from; and how both the knowledge itself; and where they get if from contributes to their contraceptive care seeking behaviour. | Can you tell me what you know about relationships, sex and/or becoming pregnant?   - what do you think are the right circumstances for a woman to become pregnant? - What do you know as the ways in which a woman can become pregnant or prevent herself from becoming pregnant? - How do you think being in a relationship could contribute to wanting/not wanting to become pregnant?   How knowledgeable do you feel about issues that have to do with sex and what should happen to become pregnant or not become pregnant? Can you tell me what you know?  Can you tell me about relationships, sex and/or becoming pregnant when it comes to your own life and relationship (s)?   - What are your thoughts (if any) about becoming pregnant? What do you think should happen for a woman to be ready to start having children? - Are you in a sexual relationship; and if so,   - how do you feel about having a child with your partner? What/How do you and your partner talk about when it comes to becoming pregnant?   - Can you describe what you do or would do to prevent getting pregnant before you are ready?   *(If already has children)* Can you tell me about becoming pregnant with your children? Did you feel ready to become pregnant- why or why not? How do you prevent becoming pregnant before you are ready?  *So, you have told me a lot about what you think and know about relationships, sex, and pregnancies,*   - - Whom or what or where do you get information about these issues?   - Whom or what are the most important sources of information to you? How did you determine the importance of each source?   - Do you rely on these information sources as facts? Which source of information do you believe the most or always trust that what they are telling you is true? Why? | It’s important to set the tone; and get a general understanding  of how young women situates pregnancy and contraception within their wider understanding of fertility and choices. |
| **Young women's knowledge and experiences of preventing pregnancies, and contraceptive use?** | We would like to understand young women's experiences of using contraceptives.  we would like to know and understand what young women know about family planning/preventing pregnancies/birth, including their fears or challenges, myths, and misconceptions around pregnancy, family planning and contraceptive use and need; | Which forms of birth control or ways of not becoming pregnancy have you used or do you know about? (   - what, why, when, and how, was the use (or non-use) of each of these ways?   Have you ever heard about contraceptives/ ‘birth controls’? - *‘control’:COC? ‘Secure’:POP? ‘jadelle/implanon’: implants? ‘Depo’: injectable? ‘loop’/IUCD? Condoms?* Can you tell me everything you know about these? Can you tell me about any other methods or ways that you know of preventing or stopping a pregnancy from happening?   - how do they work to prevent pregnancy? how long do they work to prevent pregnancy? - where do you/young women get their contraceptives from? - who can/should take contraceptives? (sexually active? married women/ sex workers? any woman?) - who (if anyone) should know that you are taking contraceptives - any side effects of contraceptives? - any fears or challenges you know about taking contraceptives   Have you ever used any of these contraceptives or any way of stopping/preventing a pregnancy?  **YES:** Can you describe to me your experience using contraceptives *(experience of each method if many have been used)* What made you start taking contraceptives? How did you decide to start taking contraceptives? what contraceptives do/did you use? Why where you using contraceptives? Did you experience any side effects or unexpected effects- tell me about that? Did you ever stop using them- can you tell me what made you stop?  **NO:** Do you know anyone who’s ever used contraceptives? YES: What do you know about their experiences/ did this change your understanding of family planning? How?  Where do you/did you get all this information (above) that you gave me about contraceptives?  if you have questions or want to know more about contraceptives, who told/tells you about contraceptives, their use and side effects (is it your parents? friends? health providers? CHIEDZA? church? school? other programs?) | We want to get an understanding of what young women know or are doing around their ‘birth control’ aka not becoming pregnant until they are ready/want to.  some of the previous findings in CHIEDZA, and other countries show that many of the young people (and some providers) in CHIEDZA, think (or speak of) contraceptive use:  1) as being for 'married' people only/situations sexual active status is implied/obvious  2) results in infertility and/or difficulty in conceiving  we want to interrogate further where young people get this kind of information from, why they believe these things as fact and how this belief contribute to their contraceptives care seeking behaviour. |
| **Topic 1b: Young women's perceptions and beliefs about becoming pregnant (or not), contraceptives, myths and misconceptions, and side effects.** | | | |
| **Young women's perceptions and beliefs around contraceptive use** | we would like to know and understand what young women know about family planning, including side effects, fears/challenges, myths, and misconceptions around family planning and contraceptive use; where they get this knowledge from; and how both the knowledge itself; and where they get if from contributes to their contraceptive care seeking behaviour  we would like to know how participants have ever taken measures to avoid, prevent or terminate a pregnancy or knows of different ways that her peers have done so. What kinds of things has she heard about and how does she think they work? | Have you ever heard /seen that using contraceptives *(the ones that we talked about above for example)* make it hard/difficult to have children/fall pregnant when you now want to?   - **YES:** Can you tell me what you heard about this? where/how did you hear about this information? Do you think it is true- why or why not? Does this influence whether or not you use contraceptives? - **NO:** what do you think about this statement? Do you think it is true- why or why not?   Have you ever heard that only married women can/should take contraceptives?   - **YES:** Can you tell me what you this you heard about this? Do you remember when in your life and how you heard about this? Where did you hear of this information? Do you think it is true- why or why not? does this influence whether or not you use contraceptives?   - Do you think unmarried women who are sexually active should take contraceptives? How do you think unmarried women should avoid or prevent pregnancies? - **NO**: what do you think about this statement? Do you think it is true- why or why not?   - Do you think unmarried women who are sexually active should take contraceptives? How do you think unmarried women should avoid or prevent pregnancies?   Can you describe how important you think it is for sexually active women to use contraceptives?  Can you describe what impact using contraceptives can have on your life? your community? | some of the previous findings in CHIEDZA, and other countries show that many of the young people (and some providers) in CHIEDZA, think (or speak of) contraceptive use:  1) as being for 'married' people only/situations sexual active status is implied/obvious  2) results in infertility and/or difficulty in conceiving  we want to interrogate further where young people get this kind of information from, why they believe these things as fact and how this belief contribute to their contraceptives care seeking behaviour |
| **Topic 2: Experiences of youth-friendliness in CHIEDZA in relation to Family planning service delivery** | | | |
| **The experiences of young women who access family planning services at CHIEDZA** | We would like to understand young women's experiences of accessing family planning services at CHIEDZA, particularly how they process and identify quality service delivery. We would like to hear from the young women, what they like or do not like about this service delivery model | Can you describe for me what happens when you come to access family planning services at CHIEDZA?   - what makes/made you come for family planning services? - what happens from the moment you arrive at the CHIEDZA centre until you leave? - What happens in the consultation booth? Do you move from one booth to another? Do they change health providers who serve you? - Do you always receive all the information you need about family planning and the contraceptives available? can you give me an example of the kinds of information you have been given before? - Have you always been able to get the contraceptive choice you wanted?   - **YES**: can you describe/tell me about what made you choose the contraceptive product you took up? Has this stayed the same or has this changed- tell me about why that is?   - **NO:** can you describe what happened when you were not able to get your contraceptive of choice? why were you not able to get it? Did you get something else?   **For Young women who took up LARCS at CHIEDZA:**   - can you tell me (how/why) what made you take up LARCS (implants, Depo, IUCD) instead of the short acting methods (oral contraceptives? - can you describe for me what happened on the day you came to get your family planning-LARCs? - Had you used oral contraceptives before? if so,   - can you tell me when you switched to a XX (LARC) and what made you make the change?   - From your experience, which do you prefer, the short-acting (pill) or the LARC you have now? why or why not? - **For implants/IUCDs**:   - can you describe what happened when you got to the nurse (PsZ) who inserted your XX? Did you feel any different from when you talk to the CHIEDZA nurse/other providers? Was it the same?   - What do you think about this process of moving from one booth/provider to another and back again to get your LARCs? How long does it take? what did you do whilst you were waiting?   What do you like about the whole family planning service delivery process at CHIEDZA? What don't you like? what don't you like? | some of the previous findings in CHIEDZA, show that many of the young people at CHIEDZA think that CHIEDZA is providing quality youth-friendly services for young people. This makes the young peopl comfortable and open up about their personal and sexual lives when they come to seek services at CHIEDZA, particularly for family planning services. However, there is not enough understanding of which parts or components of quality youth-friendly care draw young people in to access family planning services. |
|  | we would like to understand the provider aspects of youth-friendliness for family planning services in CHIEDZA | Can you describe how the providers at CHIEDZA treat you? Do you think they are youth-friendly? do not have an attitude?   - what does youth-friendliness mean to you? How are CHIEDZA providers youth-friendly (or not)? Can you give me an example of when a provider has been friendly in the way they provide family planning services for you? - when you say providers have/should not have an attitude- what does that look like for you when you are getting family planning services? Can you give a   How do you feel about the way the CHIEDZA providers treat you when you are getting family planning services? How is this different | some of the previous findings in CHIEDZA, show that many of the young people at CHIEDZA think that the providers at CHIEDZA are non-judgemental and have no attitude. This makes the young people comfortable and open up about their personal and sexual lives when they come to seek services at CHIEDZA, particularly for family planning services. However, there is not enough understanding of what it means or looks like to be 'non-judgemental' and/or have 'no attitude'. We want examples of what this is from the young people, to better discern if this is something about the personal characters of the providers; or if it's relative to care they have received elsewhere and/or a combination with other factors |
| **Topic 3: COVID-19 effects on family planning/contraceptive experiences** | | | |
| **COVID-19 impacts on family planning experiences of young women at CHIEDZA** | young women and providers have talked about how the closure of CHIEDZA in April-May 2020 reduced access to free and readily available contraceptives, which resulted, among other things, in young women 1) looking for alternative less readily available options, 2) changing contraceptive methods? we want to further understand the impact of COVID-19 on family planning experiences of young people, one year into the pandemic | Can you tell me how COVID-19 impacted your family planning/contraceptive use?   - How/where did you access contraceptives during the lockdown in 2020 (when CHIEDZA was closed and also when CHIEDZA reopened) and/or this year (when CHIEDZA remained open?   - did you face any challenges? if so, can you tell me about them?   - did you go and access your contraceptives elsewhere? can you tell me about where this is? How was it different or the same to CHIEDZA?   - did you change your contraceptive method because of the lockdown? If so, how did you like your new methods? Did you remain on that method or you went back to the method you were using before COVID-19?   - Did you just stop taking contraceptives during the lockdown? If so, how did you feel about this? - during the lockdowns: where you able to move about to go and get contraceptives from CHIEDZA or elsewhere? - In your opinion (or from what you saw in your community), how did young women access contraceptives during the lockdown? | we have initial findings on covid-19 impacts on family planning and want to continue to understand these impacts; including understanding if these impacts and/or contraceptive care choices made because of COVID-19 have continued, remained or changed. |
| **Topic 4: Young women living with HIV's experiences and use of family planning services in CHIEDZA** | | | |
| **The family planning experiences of young women living with HIV who access CHIEDZA services** | We would like to understand contraceptive choice; use and experience of young women living with HIV who access family planning services at CHIEDZA. | In your opinion, do you think your HIV status, treatment and care influenced (s) your contraceptive choice?   - if so, can you describe for me how? - if not, how might HIV status, treatment and care influence contraceptive choice?   Do you think your HIV status influences your contraceptive use?   - if so, can you describe for me how? - if not, how might HIV status, treatment and care influence contraceptive use.   Do you think your HIV status influenced (s) your family planning service provision at CHIEDZA? If so,   - If so,   - can you describe how?   - can you describe for me what happens when you come to access family planning and/or services at CHIEDZA? Do you get these services together or come separately for them? - if not, how might HIV status, treatment and care influence contraceptive use. | we want to better understand the interactions, if any, in the care-seeking behaviours for HIV and family planning for young HIV+ women; and how the CHIEDZA service delivery model supports this (or not) |

We’ve now reached the end of our discussion. We appreciate your willingness to talke and participate in this discussion with us. I will stop recording us now.

**[*Interviewer should answer any unanswered questions, and provide references as needed or clarify any* *misconceptions at this tim*e]**
